# Scalable acoustic virtual stirrer for enhanced interfacial enzymatic nucleic acid reactions

**DOI:** 10.1126/sciadv.adt6955

**Published:** 2025-03-05

**Authors:** Dayang Li, Kunjie Li, Jianquan Li, Dongfang Li, Heng Chen, Sen-Sen Li, Chaoyong Yang, Huimin Zhang, Lu-Jian Chen, Xuejia Hu

**Affiliations:** ^1^ School of Electronic Science and Engineering, Fujian Key Laboratory of Ultrafast Laser Technology and Applications, Xiamen University, Xiamen 361012, P. R. China.; ^2^ Key Laboratory of Spectrochemical Analysis and Instrumentation, Ministry of Education, State Key Laboratory of Physical Chemistry of Solid Surfaces, Department of Chemical Biology, College of Chemistry and Chemical Engineering, Innovation Laboratory for Sciences and Technologies of Energy Materials of Fujian Province (IKKEM), Xiamen University, Xiamen 361102, China.

## Abstract

Enzymatic nucleic acid reaction is a fundamental tool in molecular biology. However, high-complexity enzymatic DNA reactions and assays are still challenging due to the difficulties in integrating and scaling up microscale reaction units and mixing tools. Here, we present scalable acoustofluidic platform featuring acoustic virtual stirrer (AVS) arrays, serving as stirrers to increase the efficiency of interfacial enzymatic nucleic acid reactions. Analogous to magnetic stirrers, AVS arrays perturb the fluid through oscillating pressure nodes, controllable in terms of speeds and amplitudes via modulation. By optimizing the kinetics of surface-tethered DNA and enzymes via AVS, we achieve a 7.74% improvement in the stepwise yield of enzymatic DNA synthesis. In addition, the AVS enhanced DNA logic gate architecture can complete responses within 2 minutes, achieving average speed enhancement of 8.58 times compared to the non-AVS configuration. With its tunability, ease of integration, and efficiency, this technology holds promises for applications in biology and chemistry.

## Teaser

Scalable acoustofluidic virtual stirrer enhances the efficiency of enzymatic nucleic acid reactions.

## INTRODUCTION

Enzymatic nucleic acid reactions have become indispensable tools in molecular and synthetic biology due to their remarkable specificity and superior catalytic ability (*1*, *2*). They play a pivotal role in advancing many cutting-edge application areas, including information storage based on enzymatic DNA synthesis (*3*, *4*) and enzyme-assisted DNA computation (*5*). Most work on DNA information storage and DNA computation relies on manual implementation of in vitro carriers (*6*). However, this labor-intensive and error-prone approach to liquid manipulation struggles to meet the exponentially increasing demands of Moore’s Law for DNA-based information processing. Microfluidics has been developed for on-chip DNA information storage and DNA computation due to its unique advantages of high throughput, automation, parallelism, integration, and low agent consumption (*7*–*9*).

Typically, the fluid flow state in a microfluidic device is laminar and is dominated by viscous forces rather than inertial forces (*10*), with low Reynolds numbers in the channel and mixing mainly achieved by the diffusion of molecules across the interface. This leads to challenges in sufficient mixing, leading to direct impact on the reaction at the interface. However, enzyme-dependent reaction systems often require stringent experimental conditions, and DNA probes immobilized on the chip surface are hindered by surface crowding effects, leading to lower reaction efficiencies and severely limiting their practical application (*11*, *12*). Optimizing and improving the conformation and adsorption/desorption kinetics of surface-tethered DNA, as well as enhancing bioturbation and enzyme turnover efficiency at microfluidic interfaces, can improve the elongation efficiency of enzymatic de novo DNA synthesis and the speed of enzyme-assisted DNA logic calculations.

Various types of passive and active mixing methods have been proposed previously (*13*). For passive methods, a predefined obstacle structure or configuration of fluid channels is primarily used to induce chaotic advection, which requires continuous-flow driven by an external pump (*14*). Although passive mixers are widely adopted, there are some inevitable drawbacks that limit their practical application, including the need for complex equipment, the inability to be applied to static synthesis scenarios, and the resulting waste of reagents (*15*–*18*). Alternatively, active microreactors, which allow on-demand activation by electric (*19*), thermal (*20*), or magnetic field (*21*, *22*), have been developed to improve mixing performance. In current strategies, responsive components are usually needed in the medium, complicating the manufacturing process and necessitating the use of external equipment. Besides, high energy density caused by electric or thermal fields may introduce concerns in biocompatibility. Recently, noncontact and biocompatible acoustic waves combined with fluid dynamics have seen considerable progress, known as acoustofluidics. Also, acoustofluidics have serve applications in many fields, like biomedical applications (*23*), including particle and cell manipulation (*24*–*28*), drug loading and delivery (*29*, *30*), fluid manipulation (*31*), and biochemical analysis (*32*, *33*). Furthermore, microreactors driven by acoustics exhibit high efficiency and excellent performance, for example, micromixers through streaming effects induced by steep velocity gradients near boundaries or by the attenuation of nonlinear acoustic propagation (*34*). However, limitations exist for these methods in terms of applicability and uniformity, for instance, the need for ultrasound responsive microstructures and uneven vortex velocity in large areas due to the uniform attenuation of acoustic energy. Furthermore, considering the requirements of parallel sample processing, it is challenging to miniaturize and integrate these approaches while ensuring precise individual reaction unit control. Therefore, there is still great need for a noncontact and effective technology that is scalable to future automated solutions for driving on-chip reactions.

Here, we propose an acoustic virtual stirrer (AVS) platform that, similar to macroscale stirrers, uses acoustic waves to drive the controllable oscillation of acoustic nodes, thus constructing a virtual stirrer array within microfluids. This platform provides flexible control of the size, amplitude, and speed of oscillation based on the acoustic field’s period, input power, and hopping frequency adjustment. By lithography, the micromixer based on AVS can serve as a scalable unit adaptable for both large-scale and micrometer-scale applications. In a medium, the oscillating acoustic node lattice generates dynamic radiation forces and Eckart streaming, creating turbulence for efficient in situ mixing and mass exchange. We describe the construction of three efficient microfluidic enzyme–nucleic acid reaction systems based on AVS, including polymerization by DNA polymerase I, digestion by deoxyribonuclease (DNase) I, and ligation by T4 DNA ligase. In addition, AVS is used to enhance the stepwise yield of on-chip single-base enzymatic oligonucleotide synthesis and the computational speed of on-chip DNA Boolean logic gates. The high programmability and broad applicability of this acoustic stirrer are expected to drive advances in the use of DNA for future information technology and biomedical applications.

## RESULTS

### Working concept of AVS array

**Fig. 1.**
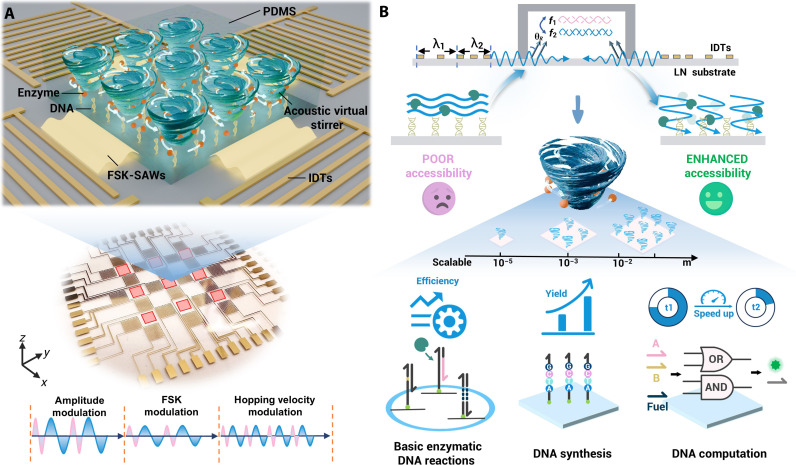
Schematic of the integrable and scalable AVS platform. (**A**) The AVS array micromixers feature two pairs of dual-wavelength IDTs, which generate oscillating acoustic fields that create vortex array in the fluid. (**B**) Without acoustic stirring, the accessibility of enzymes and primers in chambers is limited. The AVS enhances the accessibility of enzyme and substrate at the interfaces by generating turbulence. Furthermore, AVS-based microstructure-free mixing and enhanced interfacial enzymatic nucleic acid reactions are achieved.

The scalable acoustofluidic platform is illustrated in Fig. 1A. Each unit consists of two pairs of orthogonal dual-wavelength interdigital transducers (IDTs) that generate two sets of surface acoustic waves (SAWs). Upon applying radio frequency (rf) signals to the IDTs, two sets of Rayleigh waves are generated via the piezoelectric effect. These superimpose at the center of the cavity to form surface standing acoustic waves (SSAWs) (*35*, *36*). The acoustic nodes are dynamically modulated through programmable frequency shift keying (FSK) signals, resulting in the in situ formation of oscillating vortex array. Because of the gradient of the Gor’kov potential, by the interaction of acoustic radiation force (*37*), gravity, and streaming effects, fluid in the field will be relocated. Periodic switching between two frequencies results in the oscillation of the nodes, which can function as a virtual stirrer, acting on nonuniform or uniform liquids.

Figure 1B shows a side view of the unit presented in the *x*-*z* plane. In the laminar flow state, the accessibility of enzymes and initiators anchored at the interface is restricted. Consequently, the in situ vortices generated by the AVS array enhance the accessibility and affinity of enzymes and substrates. As shown below this scheme, we demonstrate multiple functionalities using the AVS platform, including homogeneous mixing without microstructures, enhancement of three typical single-step enzyme-mediated molecular biology tools, improvement of multiple iterative cycles of enzymatic DNA synthesis, and enhancement of enzyme-driven DNA computation for real-time detection. The AVS platform exhibits remarkable performance in enabling efficient microfluidic reactions.

### Interaction between acoustic field and nonuniform liquids

**Fig. 2.**
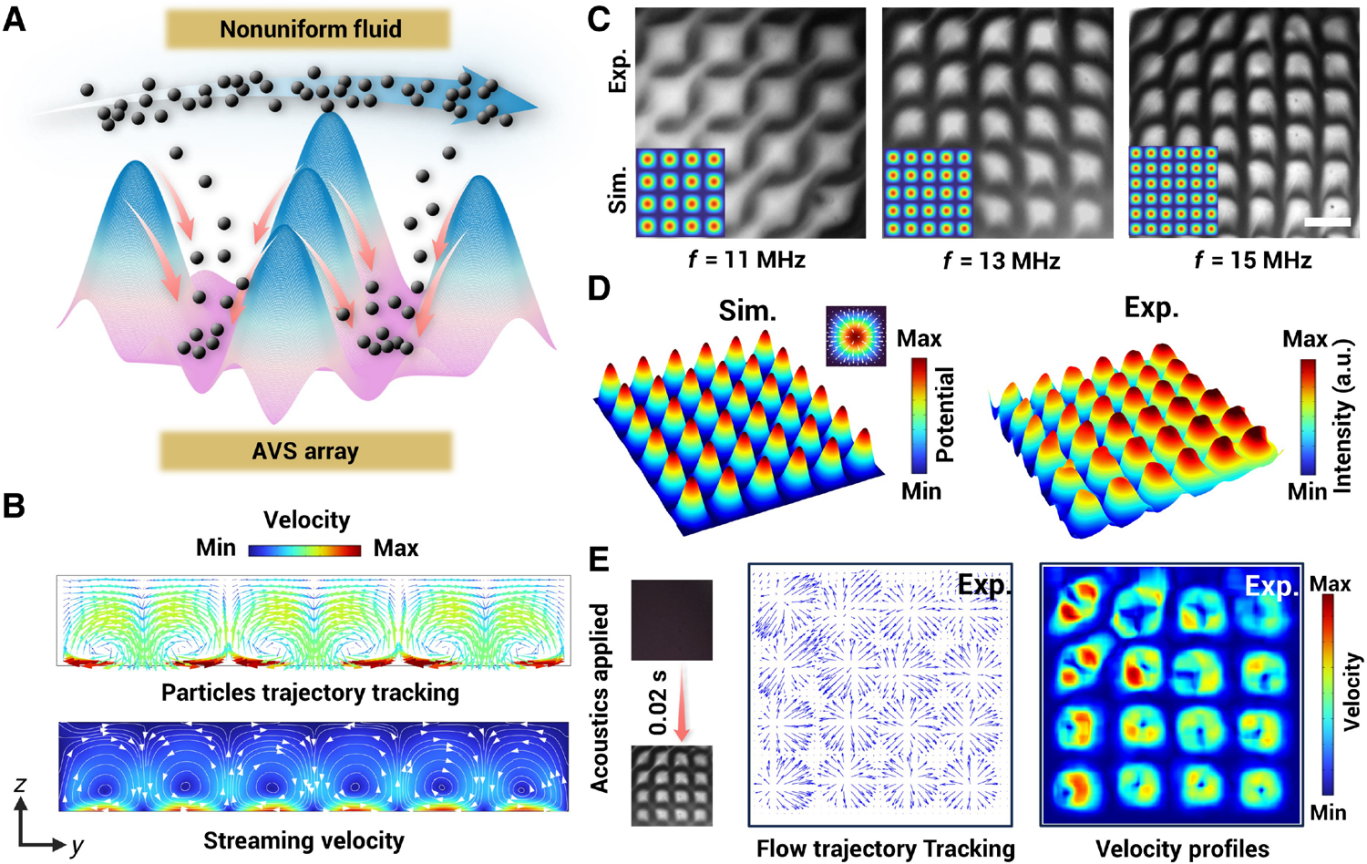
Interaction between liquid and acoustic field. (**A**) AVS array induce inhomogeneous fluid relocation and mixing. (**B**) Simulation of acoustic streaming vortex of the fluid induced by the acoustic energy attenuation. (**C**) Simulation results illustrate the acoustic field distributions at different frequencies, along with the corresponding experimental results of the reconstruction and reconfiguration of nonuniform fluid by SSAWs (11 MHz, 13 MHz, and 15 MHz). (**D**) Comparison of simulated and experimental results for the SSAW field and fluid concentration field. a.u., arbitrary units. (**E**) Images are captured before and after acoustics are applied and are used for tracking the flow trajectories and velocity profiles. The flow field at the moment acoustics are applied is analyzed using image algorithms. Scale bar, 150 μm.

In Fig. 2A, the schematic shows how nonuniform fluids are affected by SSAWs, where inhomogeneous fluids within the field experience a gradient acoustic radiation force (*38*, *39*). Liquids with higher acoustic impedance tend to move to pressure nodes (PNs), and the lower ones tend to move toward pressure antinodes (ANs). Dynamic oscillating acoustic nodes function as a stirrer, driving the fluid moving forward and back, until the miscible liquid is mixed into a homogeneous phase by convection-diffusion. In addition to the radiation force, an acoustic streaming effect originating from the attenuation of propagating acoustic waves in the medium will also contribute to the stirring effect. As depicted in Fig. 2B, numerical results describe the periodic distribution of acoustic streaming vortices in the *x*-*z* plane, as well as particle trajectory tracking. The streaming rises from the ANs on the substrate and rotate clockwise or counterclockwise at the adjacent PNs (*40*).

To validate the interaction between acoustics and liquid, experiments were conducted in a microfluidic chip. Microfluidic pumps were used to inject a dye solution and deionized water into the chip at a constant flow rate (as shown in fig. S1), and an AVS micromixer unit was used to conduct the whole area mixing. By varying the frequencies of acoustic waves applied in two orthogonal directions, the acoustic potential field is regulated to control the patterning of nonuniform fluids. As shown in Fig. 2C, the simulated acoustic potential fields are at activation frequencies of 11, 13, and 15 MHz. These exhibit distinct periodicities, which correspond closely to the periodic square lattice structures observed experimentally at the interface between the two fluids. In the left panel of Fig. 2D, the simulated acoustic potential field is presented at a frequency of 15 MHz, with an inserted image illustrating the distribution of radiation force directors near ANs. Given that the concentration of a dye solution correlates with the grayscale intensity in bright-field images, the right panel of Fig. 2D presents a normalized grayscale intensity color map of the fluid, derived from the experimental data shown in Fig. 2C. This color map aligns well with the simulations, revealing that the nonuniform fluid undergoes rearrangement under the influence of the acoustic field. Specifically, fluid of high concentration accumulates in the regions of lowest potential, whereas fluid of low concentration is found in the regions of highest potential. To visualize the dynamic process of fluid rearrangement in the SSAW field, an optical flow tracking algorithm is used to analyze experimental images from two adjacent frames after acoustics are applied to obtain the migration velocity vector distribution as shown in Fig. 2E. The fluid is rapidly pushed toward PNs from ANs, by both the acoustic force and the streaming effect, and is consistent with the simulations. Vorticity profiles during acoustic streaming reconstruction are further obtained based on this analysis.

Thus far, the acoustic nodes in the SSAW field can be used for fluid manipulation in microstructure-free microfluidic cavities, forming the fundamental working mechanism for realizing an AVS unit.

### Spatiotemporal dynamics and modulation of AVS

**Fig. 3.**
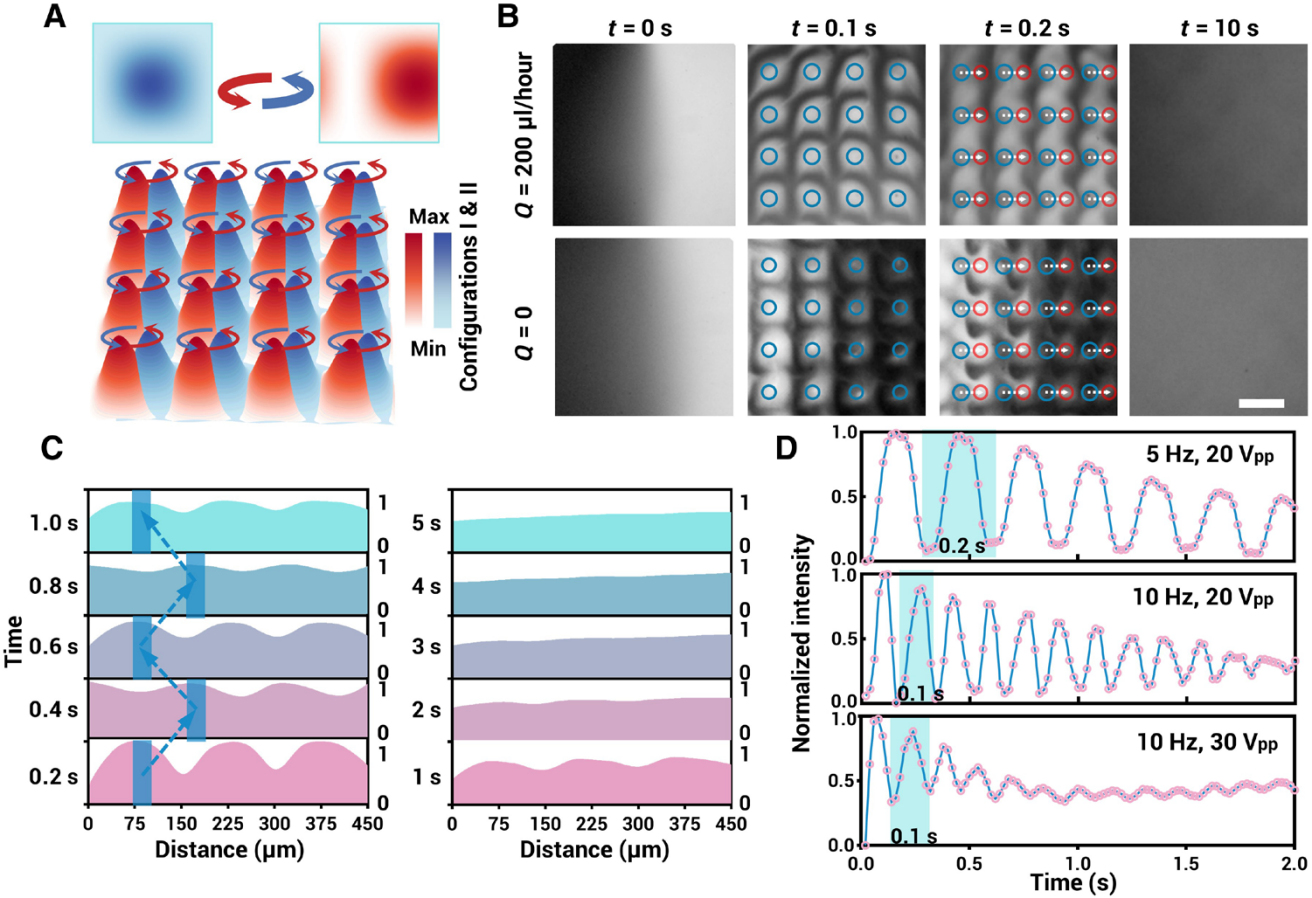
Dynamic oscillating AVS for fluid mixing. (**A**) Simulation results of the acoustic field and oscillating ANs under two different configurations. (**B**) Experimental results of fluid distribution and the mixing process induced by AVS array at *t* = 0, *t* = 0.1 s, *t* = 0.2 s, and *t* = 10s. (**C**) Normalized gray value profiles along the white lines in (B) are measured at various time points, whereas the AVS array oscillates at 5 Hz. (**D**) Curves of normalized gray value variation over time in the circular area in the fluid are plotted for different acoustic parameters. Scale bar, 150 μm.

The acoustic micromixer unit is designed with two finger spacings across the width of the transducer electrodes, enabling excitation by dual frequencies and generating two spatially periodic acoustic potential fields. The unit consists of 10 pairs of electrodes with 75-μm finger spacing and 10 pairs of electrodes with 65-μm finger spacing. As shown in Fig. 3A, the results of the numerical simulation illustrate the superposed acoustic fields under dual-frequency excitation. Configuration I corresponds to 13 MHz, and configuration II corresponds to 15 MHz. When IDTs in orthogonal directions simultaneously apply modulated FSK signals, each PN undergoes stable and regular periodic oscillations. The oscillation capability of this strategy is demonstrated using 12-μm-diameter fluorescein isothiocyanate–labeled polystyrene (PS) microspheres. (The dynamic process under different oscillation speeds is recorded in movie S1.)

Building on this, an oscillating AVS array is constructed on chip. When injecting two liquids into the microfluidics chip, AVS can effectively mix the two miscible liquids, as shown in Fig. 3B. In the experiments, the mixing process is recorded when the hopping frequency (*f*_FSK_) is set to 5 Hz and voltage is set to 20 V_pp_. The AVS array causes the concentration field to initially distribute in a grid pattern. After half a cycle, the grid points quickly move nearby and continuously cycle until the concentration is uniformly distributed. After 5 s, the liquid is totally mixed by the enhanced convection-diffusion process (a representative mixing process in the entire microfluidic chamber is recorded in movie S2). The grayscale intensity profiles along a line (450 μm long) in the chamber center are analyzed to quantitatively visualize the mixing process (Fig. 3C). From this analysis, the normalized gray value peaks obviously shift regularly at a certain amplitude, constantly moving back and forth with oscillating acoustic nodes (5 Hz). In addition, the gray value distributions become smooth after several seconds, leading to a homogeneous liquid distribution. This acoustic stirrer is quite flexible in controlling the process. For example, the *f*_FSK_ can be adjusted from 10^−2^ to 10^3^ Hz, and the applied signal voltage can also be tuned, corresponding to the oscillating speed and amplitude of AVS. In Fig. 3D, the normalized data of the average gray value within one of the blue circles in Fig. 3B are monitored and recorded when different acoustic parameters are applied. It can be observed that the curves continue to oscillate periodically and gradually flatten as the periods match the *f*_FSK_. In addition, the curves flatten out faster when the amplitude reaches 30 V_pp_.

### AVS for fluid mixing

**Fig. 4.**
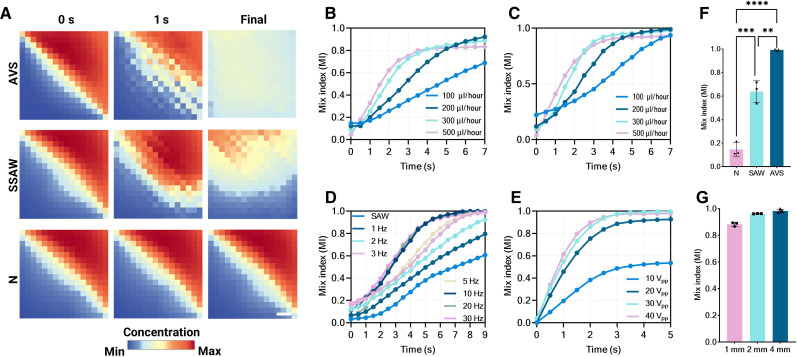
Characterization of the mixing performance of tunable AVS. (**A**) Experimentally observed heatmaps of fluid concentration distribution at *t* = 0, *t* = 1 s, and final states. (**B** to **E**) Characterization of MI in the reaction region under different excitation voltages, flow rates, and oscillation speeds. (**F**) Comparison of the MI of control, SSAW, and AVS groups (**P < 0.01; ***P ≤ 0.001; ****P ≤ 0.0001). (**G**) Characterization of the MI in different devices (chambers with different sizes). Scale bar, 500 μm.

Mixing is typically achieved using the acoustically driven stirrer with the working scale ranging from micrometers to millimeters. Black dye solution and deionized water are injected into a microfluidic chamber via syringe pumps. Figure 4A shows dye concentration fields extracted and plotted as a heatmap. In the absence of agitation, the fluid exhibits distinct laminar flow characteristics due to the continuous flow field (*Q* = 200 μl/hour), and a clear interface can be observed (N group). When the SSAW field is applied without the FSK modulation (SAW group; 20 V_pp_), although the laminar flow is disrupted by acoustic nodes, the stirring effect is absent. As a result, it is not possible to achieve a homogeneous state. By using FSK modulated signals (AVS group; 20 V_pp_, *f*_FSK_ = 5 Hz), the AVS generates a stirring effect and induces chaotic flow, leading to direct cross-mixing of the two-phase flow.

To quantitatively assess the mixing capacity of AVS dependence on input power, oscillation speed, and flow velocity, the mixing index (MI) is calculated and normalized (see note S3). At an input power of 20 V_pp_ and a hopping frequency of *f*_FSK_ = 1 Hz, the mixing coefficients versus time for different flow rates are shown in Fig. 4B. At low flow rates (100 μl/hour), the actual mixing coefficient (68%) is lower compared to the high flow rate case (92 and 88% for flow rate of 200 and 300 μl/hour, respectively). This may be explained by continuous flow mixed by virtual acoustic pillars, in which the oscillating ANs can function as virtual pillars to induce fluid separation and convective mixing. This effect can obviously contribute to the increase in MI when *Q* increases. However, with a continuous increase in *Q* to 500 μl, the MI decreases, as indicated by the curves. In Fig. 4C, the MI at different *Q* values is presented when *f*_FSK_ is increased to 30 Hz. The AVS demonstrates much faster mixing under these conditions, and more than 90% MI can be achieved in less than 4 s. Figure 4D shows the stirring effect as a function of hopping frequency and signal amplitude. The MI increases over time when adjusting *f*_FSK_ from 0.5 to 30 Hz, with a constant flow rate of 300 μl/hour and input power of 20 V_pp_. These results indicate that higher oscillation speeds of AVS result in increased mixing efficiency. In addition, Fig. 4E shows the measured MI curves when applying different rf voltages (set *f*_FSK_ to 30 Hz and *Q* to 300 μl/hour). When the voltage increases from 10 to 20 V_pp_, the final MI can increase from 53% to more than 90%. By continuously increasing the voltage to 40 V_pp_, the fluid in the chamber can reach a higher MI and experience much faster mixing speeds.

Figure 4F presents a quantitative comparison of the MI between the AVS, SSAW, and control groups. For the control group, only 14% MI is achieved through the diffusion process. In the SSAW group, because of the virtual pillar effect of the ANs as previously mentioned, the final MI increases to 63%. However, when the AVS is applied, the MI reaches more than 99% (10-Hz *f*_FSK_ and 20-V_pp_ voltage), indicating that this active strategy is highly efficient. In addition, this unit can be easily scaled and can be integrated with different reaction platforms. Figure 4G verifies the effectiveness of the AVS in microchambers of different sizes (1, 2, and 4 mm), with final MI values of 88, 96, and 98% achieved for these cavities, respectively.

In summary, the in situ mixing efficiency of the AVS unit is governed by the interplay of oscillation speed, input power, and flow rate. With these tunable parameters, the AVS platform is quite suitable for the enhancement of various chemical and biological reactions.

### AVS for enhanced interfacial enzymatic nucleic acid reaction

The constructed AVS was used to enhance the efficiency of interfacial enzymatic nucleic acid reactions. In particular, we optimized the AVS platform in terms of temperature control and interface modification to better match these reactions. Precise temperature control is crucial for enzyme activity, stability, and efficiency. An additional temperature control device increases system complexity and redundancy. Here, by leveraging the acoustothermal effect produced by the viscoelastic damping of polydimethylsiloxane (PDMS) under an acoustic field, we constructed an acoustothermal control module superimposed on the AVS, allowing temperature control simply by adjusting the amplitude of the applied ac voltage (*41*). The dynamic temperature control process of the liquid inside the PDMS center using the AVS was investigated with an infrared thermal camera. The results show that the temperature can be precisely controlled through input power (fig. S2A), with excellent stability and reproducibility (Fig. S2B). Furthermore, the temperature SD for the actual reaction area (2 mm) decreases by 84.5% compared to the entire central heating area (4 mm) (fig. S2C). Besides, virtual stirring enhances fluid convection and mixing within the chamber, further improving the accuracy and uniformity of the temperature distribution in the reaction area. The temperature variation within the AVS working area remains below ±0.8°C from the set steady-state temperature (fig. S2D), which is acceptable for enzymatic reaction efficiency (*42*–*44*). On the other hand, to establish an in situ enzyme-DNA reaction platform on the AVS, it is necessary to achieve uniform and robust DNA modification at the interface. Unlike glass substrates, lithium niobate’s highly ordered crystal structure and high surface energy make it prone to forming a stable oxide layer, complicating surface modification (*45*). To address this issue, we used a “radical activation–silanation–amidation” strategy to modify single-stranded DNA with an amino group at the lithium niobate interface. The hybridization results of the complementary fluorescent strands validate the feasibility of this strategy (fig. S3). In addition, poly(amidoamine) (PAMAM) dendrimer was introduced to enhance the density of modified anchors at the interface (*46*). Fluorescent results showed that the density of DNA modified at the interface was significantly enhanced by 114.1%, providing an important basis for acoustofluidic in situ enzyme-DNA reactions.

**Fig. 5.**
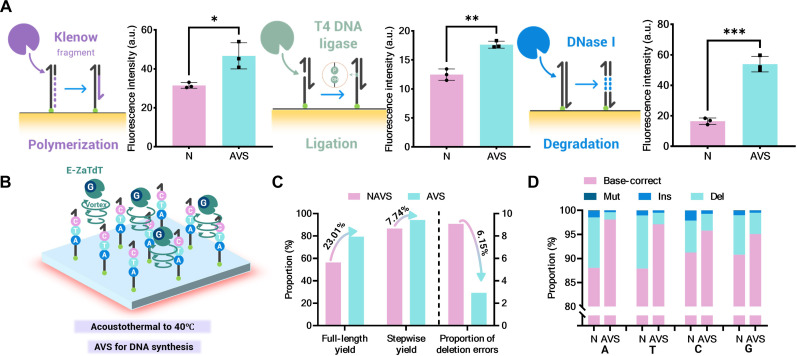
Enzyme-catalyzed DNA reaction on the AVS interface. (**A**) Schematic diagrams depicting the polymerization catalyzed by DNA polymerase I, the ligation catalyzed by T4 DNA ligase and the digestion catalyzed by DNase I, along with comparisons of interfacial reaction efficiency when using AVS versus not using AVS. (**B**) Schematic diagram outlining the iEOS process on AVS. (**C**) Comparison of the stepwise yield, full-length yield, and deletion error rate with and without the use of AVS. (**D**) On the basis of NGS results, a synthetic step-by-step yield was determined through comparison with the target sequence (*P < 0.05; **P ≤ 0.001; ***P ≤ 0.0001).

To demonstrate the efficiency enhancement of the AVS on large-area interfacial enzymatic nucleic acid reactions, we selected three typical enzyme-mediated molecular biology tools acting on global microfluidic nucleic acids: polymerization by DNA polymerase I, digestion by DNase I, and ligation by T4 DNA ligase. We quantitatively characterized the interfacial reaction efficiency by comparing the extent of these reactions with and without the use of the AVS (AVS and non-AVS) over the same time period. Fluorescence results indicated that, with the assistance of the AVS, the efficiencies of polymerization, digestion, and ligation reactions at the interface increased by 41.4, 226.7, and 48.3%, respectively (Fig. 5A).

Subsequently, we chose interfacial enzymatic oligonucleotide synthesis (iEOS), which involves multiple iterative cycles, to further illustrate the exponential enhancement of interfacial enzymatic reaction efficiency afforded by the acoustofluidic stirrer. Here, a two-step enzymatic DNA synthesis mediated by engineered terminal deoxynucleotidyl transferase from *Zonotrichia albicollis* (E-ZaTdT) was used, building on previous studies (*47*, *48*). Unlike the magnetic bead (MB) solid-phase carriers used in earlier studies, we modified the initiator primers directly on the lithium niobate interface. We evaluated the enzymatic synthesis yield and error rate for a four-base DNA fragment (5′-ATCG-3′) under both AVS and non-AVS conditions (Fig. 5B). Over the course of four cycles, the stepwise yield of iEOS under AFS conditions reached 94.41%, which is markedly higher compared to the 86.67% stepwise yield observed under non-AFS conditions (Fig. 5C). The predominant source of iEOS errors is deletions, which primarily arise from the inaccessibility between the enzyme and the initiator primers due to the laminar flow characteristics of microfluidics. Our results show that AVS reduced the deletion error rate from 9.09 to 2.94% compared to non-AVS conditions, demonstrating that the turbulence induced by AVS within the microchamber effectively mitigates enzyme-substrate accessibility issues, thereby enhancing synthesis yield (Fig. 5, C and D).

The aforementioned reactions demonstrate the substantial advantage of the AVS in terms of end-point efficiency and yield. However, for another class of enzymatic nucleic acid reactions, such as enzyme-mediated DNA logic gates, the focus shifts to reaction rate. Now, most DNA logic gates are implemented through homogeneous reactions in tubes or bead-based solid supports (e.g., MBs) (*6*). These methods face limitations in reagent exchange, which hinder further complexity, scalability, and automation of DNA computation.

**Fig. 6.**
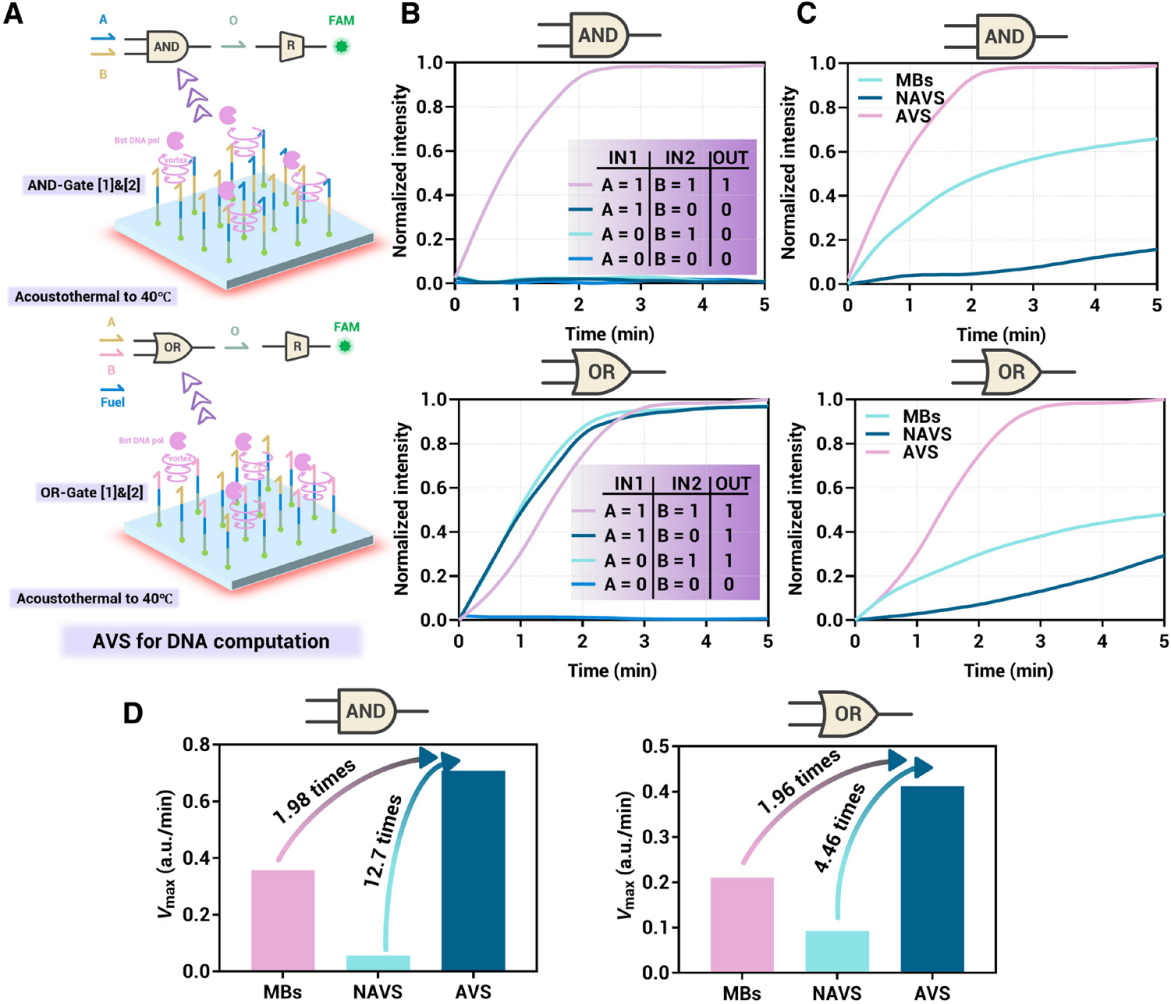
Enzyme-mediated DNA logic gate computational reaction on the AVS interface. (**A**) Schematic diagrams illustrating the logic AND and OR gates on the AVS. (**B**) Both the AND gate and OR gate were subjected to testing, and they consistently produced correct outputs in all tested scenarios. (**C**) Comparison of the execution results of the AND gate and OR gate on MBs, NAVS, and AVS, all within a 5-min timeframe. (**D**) Comparison of the *V*_max_ values for the AND gate and OR gate on MBs, NAVS, and AVS, also within a 5-min timeframe.

Microfluidics presents a promising avenue for the future of DNA computation, including the development of integrated DNA computing chips. Nonetheless, a critical challenge remains: the inherently low reaction rate of enzymatic nucleic acid reactions at the microfluidic interface, which could become the “Achilles’ heel” of DNA computing chip development. To address this issue, we attempted to construct Boolean DNA logic gates on the AVS to enhance the interfacial reaction rate by acoustically induced turbulence. Specifically, two DNA logic gates, AND and OR, based on polymerase chain displacement reactions were constructed on the AVS, building on the reported work of Song *et al.* (Fig. 6A) (*49*). The initial strands used for logical computation were modified on the lithium niobate substrate. Upon the addition of input and reporter strands, the logical output was obtained by observing and quantifying the fluorescence results at the interface. The truth value results indicated that both the AND gate and OR gate were successfully constructed on the AVS (Fig. 6B).

We also constructed the same logic gates on MBs and a microfluidic interface without AVS for comparison. Reaction temperature, enzyme concentration, and strand concentrations (input, gate, and reporter strands) were kept constant for all gates to ensure accurate comparisons. The dual truth value inputs ([A] = 1 and [B] = 1) of the AND gate and OR gate were evaluated under the three conditions (MBs, non-AVS, and AVS), respectively (Fig. 6C). From the comparison of reaction extents, AVS reached the enzymatic reaction kinetics plateau (normalized extent greater than 95%) within 2 min. In contrast, both MBs and non-AVS remained in the rising phase, requiring at least 25 min to complete the reaction (fig. S4). Considering that enzymatic reactions initially produce fluorescent reporters at an approximately linear initial rate, this closely reflects the actual reaction rate of enzymatic DNA logic gate computation. Therefore, we defined the maximum slope of fluorescence kinetics within 5 min as the maximum reaction rate (*V*_max_). Compared to MBs and non-AVS, AVS enhanced the maximum reaction rate of the AND gate by 1.98 and 12.7 times, respectively, and the OR gate by 1.96 and 4.46 times, respectively (Fig. 6, D and E). Notably, the logic gates on AVS exhibited computation speeds comparable to, or even superior to, those in homogeneous reactions, highlighting the potential for constructing more complex DNA computing systems based on this approach.

Theoretically, AVS can be arranged into multilevel arrays on LiNbO_3_ wafers through miniaturized and integrated design to perform different parallel reactions. As a proof-of-concept experiment, we developed an acoustofluidic reactor with nine independent reaction units (fig. S5A). By simultaneously or selectively activating the AVS units, reactions and detections in each region were enhanced by the acoustic virtual stirring effect. Using Boolean DNA logic gates as an example, a PDMS- and LiNbO_3_-based assembly with fluid channels and reaction zones was fabricated. The nine inputs (covering eight different input conditions for the two logic gates) were delivered to the AVS reaction zones through microchannels to initiate DNA computation. After 3 min, all nine units produced correct fluorescence outputs (fig. S5B). These results validate the scalability of the proposed AVS-based approach.

## DISCUSSION

AVS uses acoustic nodes as oscillating virtual stirrers, enabling precise and active fluid stirring at the various scales. By modulating acoustic parameters, such as oscillation speed, amplitude, and array periodicity, AVS offers flexible control over the stirring process. Unlike conventional methods that rely on microstructures or micro-obstacles to induce turbulence through continuous flow, AVS is effective across a wide range of flow rates. Experimental results demonstrate the high efficiency of this approach for both static laminar and dynamic flows. In addition, this method does not require auxiliary components, such as magnetic particles. Its implementation, achieved by integrating transducers near the mixing region, substantially broadens its applicability, including potential scenarios such as large-scale deployment, miniaturized integration, and high-throughput applications. Enzymatic nucleic acid reactions, such as enzymatic DNA synthesis and enzyme-mediated DNA logic gates, serve as examples to illustrate how AVS improves the efficiency of interfacial reactions in two critical aspects: end-point yield and reaction rate. After four rounds of single-base synthesis of the specified sequence 5′-ATCG-3′ with this device, we obtained an average stepwise yield of 94.41%, improving the cycle elongation efficiency by 7.74% compared to the free diffusion interfacial reaction. In addition, the acoustofluidic stirrer unit reduces the major error of deletion by 6.15%, demonstrating superior performance in increasing reaction adequacy in DNA synthesis experiments. AVS completes both the AND gate and the OR gate DNA logic computation in less than 3 min, ~12.7 times faster compared to free diffusion. This lays the groundwork for the construction of faster and more complex cascade circuits in the future.

## MATERIALS AND METHODS

### Design and fabrication of AVS

For the IDTs, a thin layer of S1813 photoresist (Suzhou Research Material Micro Technology Co. Ltd., China) was applied to a 128° Y-cut lithium niobate substrate (NANOLN, Jinan Jingzheng Electronics Co. Ltd., China). After transferring the electrode pattern via ultraviolet exposure, the exposed photoresist areas were removed with a developer (Suzhou Research Material Micro Technology Co. Ltd.). A thermal evaporation system was then used to deposit 5 nm of the Cr adhesive layer and 80 nm of the Au conductive layer onto the substrate. After that, the IDTs were retained on the substrate through a stripping process. For the chamber, SU-8 2050 photoresist (Microchem, USA) was coated on a silicon wafer to produce the microstructural features with 100 μm in height. The silicon wafer with chamber pattern was placed in a petri dish, and a PDMS precursor solution (10:1 of PDMS and curing agent) was poured into the mold. Following that, the petri dish was cured at 90°C for 20 min. After fully curing, the PDMS chamber layer was peeled off from the mold and immediately bonded to the lithium niobate substrate following oxygen plasma treatment to fabricate the final AVS chip.

Within the same set of IDTs, two different widths (finger width and spacing) were designed: 65 μm corresponding to a 15-MHz frequency, with 75 μm corresponding to a 13-MHz frequency. Signal generators (UTG962, UNI-Trend Technology Co. Ltd., China) and amplifiers (LZY-22+, Minicircuit, USA) were used to activate the combined IDTs and generate oscillating SAWs. The fluid in the microfluidic chamber was delivered by a syringe pump with an adjustable flow rate.

### Multiphysics simulation of acoustic field and acoustic streaming induced by AVS

A two-dimensional (2D) simulation model of the IDTs acoustofluidic platform was developed using a finite element analysis method. The model incorporates modules for laminar flow, pressure acoustics, fluid heat transfer, solid mechanics, electrostatics, and rarefied matter transfer. The acoustic potential field distribution was simulated and computed using MATLAB. Further details about these simulation models are provided in notes S1 and S2 and table S1.

### Amino modification on the lithium niobate substrate

The preparation of 3D dendrimeric and 2D amino-modified lithium niobate slides followed established protocols (*46*). For the 3D dendrimeric lithium niobate slides, thoroughly cleaned lithium niobate substrates were treated with a piranha solution (the ratio of concentrated sulfuric acid and hydrogen peroxide is 7:3, v/v) at room temperature for 2 hours. Subsequently, the hydroxyl-activated slides were exposed to a 5% (v/v) solution of (3-glycidyloxypropyl) trimethoxysilane (GOPTS) for 8 hours to introduce epoxy groups at the interface. After washing with anhydrous ethanol and drying under nitrogen, the slides were baked at 110°C for 1 hour to covalently fix the GOPTS. The silanized slides were then immersed in a 0.3% (v/v) PAMAM solution and agitated overnight (12 to 15 hours) to achieve a high density of amino-modified sites. Following modification, the slides were washed three times with methanol and dried. The 3D dendrimeric lithium niobate slides were then stored at 4°C until further use. For the preparation of 2D amino-modified lithium niobate slides, the hydroxyl-activated substrates were treated with a 5% (v/v) solution of 3-aminopropyltriethoxysilane (APTS) in anhydrous ethanol for 2 hours. After washing and drying, the slides were baked at 110°C for 1 hour and subsequently stored at 4°C until required.

The amino-terminated oligonucleotides required for the AVS enzymatic nucleic acid reactions are detailed in table S2. The amino-modified primers were attached to the lithium niobate interface as follows: The amino-rich lithium niobate slides, prepared as described above, were reacted with 1.58 M succinic anhydride (*N*,*N*-dimethylformamide containing 1% triethylamine, v/v) for 1 hour with shaking. After washing, 2.5 μM amino-modified primers to be modified was added to a 100 μM 2-morpholinoethanesulfonic acid hydrate (MES) buffer (pH = 4.5) containing freshly prepared 1-(3-dimethylaminopropyl)-3-ethylcarbodiimide (EDC) (20 mg/ml) and reacted for an additional hour with shaking. Last, a 100 mM tris-HCl buffer (pH = 7.5) was used for 30 min to remove EDC-activated carboxyl intermediates. The modified lithium niobate slides were characterized and quantified by hybridizing with a complementary strand labeled with a cyanine 3 (Cy3) fluorescent group.

In the enzymatic DNA reaction experiments, the lithium niobate substrate was prefunctionalized with amino-modified DNA strands. The PDMS chamber layer was then bonded to the substrate using the previously described method. To minimize nonspecific adsorption of biological molecules, including DNA and enzymes, a 1% (m/v) bovine serum albumin solution was introduced into the chamber and incubated for 1 hour (*50*, *51*), effectively blocking pores on the PDMS surface.

### Polymerization, digestion, and ligation on AVS

Three typical enzymatic nucleic acid reactions performed on the AVS include polymerization by DNA polymerase I, digestion by DNase I, and ligation by T4 DNA ligase. Polymerization: An annealing buffer containing a 2.5 μM complementary primer strand [20 mM tris acetate and 5 mM magnesium acetate (pH 7.6)] was added to the modified AVS. The AVS was then heated to 85°C for 2 min and subsequently cooled to room temperature to facilitate the annealing of the primer and interfacial template DNA. Following this, a 10-μl polymerization reaction buffer was prepared by mixing Klenow fragment of DNA polymerase (1 U/μl M0210, NEB), 100 μM deoxycytidine triphosphate–Cy3, 5 mM deoxy-ribonucleoside triphosphate (dNTP) mix, 50 mM NaCl, 10 mM tris-HCl, 10 mM MgCl_2_, and 1 mM dithiothreitol (DTT) (pH 7.9). This buffer was added to initiate the polymerization reaction at room temperature for 2 min. Digestion: A 10-μl digestion reaction buffer was prepared by mixing DNase I (50 U/μl; M0303, NEB), 10 mM tris-HCl, 2.5 mM MgCl_2_, and 0.5 mM CaCl_2_ (pH 7.5). The buffer was added to initiate the digestion reaction for 10 min at 37°C. Ligation: An annealing buffer containing 2.5 μM preannealed splint-adapter double-stranded hybrid was first added to the modified AVS. The AVS was heated to 85°C for 2 min and then cooled to room temperature to facilitate the annealing of the splint strands and interfacial template DNA. A 10-μl ligation reaction buffer was then prepared by mixing T4 DNA ligase (600 U/μl; N103-01, Vazyme), 5 μM phos-tail adapter-6-carboxyfluorescein (FAM), 66 mM tris-HCl, 10 mM MgCl_2_, 1 mM DTT, and 1 mM adenosine triphosphate (pH 7.6). This buffer was used to initiate the ligation reaction for 5 min at 30°C. The AVS was operated with an input power of 25 V_pp_ and an oscillation frequency of *f*_FSK_ = 10 Hz. Each enzymatic reaction was tested both with and without the AVS. Fluorescence microscopy was used to observe and quantify the changes in fluorescence before and after the interfacial reactions to assess the impact of the AVS.

### Single-nucleotide enzymatic oligonucleotide synthesis on AVS

The single-nucleotide enzymatic oligonucleotide synthesis reaction on AVS followed a well-established protocol containing three steps of extension, deprotection, and washing (*47*, *48*). Specifically, a 10-μl standard synthesis mix was prepared by mixing E-ZaTdT (0.5 mg/ml), 0.25 mM 3′-ONH_2_-dNTPs (Firebird Biomolecular Sciences), 0.25 mM CoCl_2_, 100 mM NaCl, and 50 mM phosphate-buffered saline (PBS) buffer (pH 6.8). The reaction mixture was added to the AVS, and the extension was initiated with an input power of 25 V_pp_ and an oscillation frequency of *f*_FSK_ = 10 Hz for 10 min at 40°C. Then, the interfacial products were deprotected for 1 min with sodium nitrite buffer (0.7 M, pH 5, adjusted with nitrous acid) and washed twice using 50 mM PBS buffer (pH 6.8) in preparation for the next addition cycle. All buffers used contained 0.01% (v/v) Triton X-100. These three steps form a single-base cycling process for synthesizing the target DNA sequence. Upon completion of the enzymatic synthesis reaction, the final products were recovered from the interface and prepared for next-generation sequencing (NGS) to quantitatively evaluate the synthesis (detailed steps are provided in note S4). On-chip single-base enzymatic oligonucleotide synthesis was assessed under conditions both with and without AVS.

### DNA Boolean logic gates on AVS

The construction of DNA Boolean logic gates on AVS was conducted following the methods outlined by Song *et al.* (*49*) The sequences of oligonucleotides used to assemble the OR and AND gates are provided in table S2. Each gate included two inputs (I) and one output (O), with each input encoded by a high (logical “1”) or low (logical “0”) concentration of the corresponding DNA strand. A double-stranded fluorophore-quencher reporter (R-FQ) was preannealed at a concentration of 2.5 μM before use.

For the OR gate, a 2.5 μM fuel oligonucleotide (F) was introduced and annealed with the interfacial gate DNA prior to adding the input strands. The 10-μl input mix for both OR and AND gates contained Bst DNA Polymerase (0.4 U/μl M0537, NEB), 20 mM tris-HCl, 5 mM dNTP mix, 2 mM MgSO_4_, 50 mM KCl, 10 mM (NH_4_)_2_SO_4_, 0.1% (v/v) Tween 20, and 2.5 mM R-FQ (pH 8.8). When the input was logical “1,” input oligonucleotides at a concentration of 2.5 mM were added to the mix; otherwise (logical “0”), the corresponding oligonucleotides were omitted. Following the addition of the input oligonucleotides, fluorescence microscopy was used to capture images of the interface fluorescence intensity changes at 2-s intervals, producing a series of fluorescence images. The fluorescence intensities were subsequently quantified using the ImageJ software. As for fluorescence images corresponding to a logical output of “1,” the minimum value was set to 0, and the maximum value (the average of the last three observed data points) was set to 1, after which the results were normalized and plotted. For images corresponding to a logical output of “0,” normalization was performed using the maximum value from the set of images with a logical output of “1” for the same logic gate (also the same reporter), and curves were plotted accordingly. The AVS was operated with an input power of 25 V_pp_ and an oscillation frequency of *f*_FSK_ = 10 Hz at 40°C. On-chip DNA Boolean logic gates were tested under conditions both with and without AVS. For comparison, in-tube reactions (MB-based) were conducted at 40°C using the same strand concentrations.

### Statistical analysis

Statistical analyses were conducted using GraphPad Prism 9 software, Excel, and MATLAB. The *P* value was determined by the Mann-Whitney *U* test. A correlation was considered statistically significant at *P* values below 0.05. All data are presented as the means ± SEM (*n* = 3), unless otherwise specified.

## Supplementary Materials

### The PDF file includes:

Supplementary Notes 1 to 4 Figs. S1 to S5 Tables S1 and S2 Legends for movies S1 and S2 References
