# Supplementary material for: Scalable acoustic virtual stirrer for enhanced interfacial enzymatic nucleic acid reactions
Source: Sci Adv. 2025 Mar 5;11(10):eadt6955. doi: 10.1126/sciadv.adt6955 (PMC11881892; doi:10.1126/sciadv.adt6955)
Supplement: Supplementary file 1 — Supplementary Notes 1 to 4 Figs. S1 to S5 Tables S1 and S2 Legends for movies S1 and S2 References [file sciadv.adt6955_sm.pdf]

Science Advances
AAAS

Supplementary Materials for

Scalable acoustic virtual stirrer for enhanced interfacial enzymatic nucleic acid reactions

Dayang Li *et al.*

Corresponding author: Huimin Zhang, hmzhang@xmu.edu.cn; Lu-Jian Chen, lujianchen@xmu.edu.cn; Xuejia Hu, xjhu@xmu.edu.com

Sci. Adv. **11**, eadt6955 (2025)
DOI: 10.1126/sciadv.adt6955

The PDF file includes:

Supplementary Notes 1 to 4
Figs. S1 to S5
Tables S1 and S2
Legends for movies S1 and S2
References

Other Supplementary Material for this manuscript includes the following:

Movies S1 and S2

Supplementary Notes

Note 1. Simulation of the lateral periodic distribution of acoustic streaming

To simulate the acoustic field and second order streaming generated by AVS, we used finite element method (COMSOL Multiphysics V6.2a, Stockholm, Sweden). The model dimensions were determined based on the size of the experimental device. Initially, the lithium niobate piezoelectric substrate was modeled using the "Solid Mechanics" and "Electrostatics" modules, with a low-reflection boundary applied to the bottom of the substrate. Two different potentials were applied to the IDTs. Next, the PDMS chamber was modeled using the "Pressure Acoustics, Frequency Domain" module to simulate the acoustic field, followed by solving the pressure field governed by the modified Helmholtz equation:

$$\nabla \cdot \left(-\frac{1}{\rho_c} (\nabla p_t - q_d) \right) - \frac{k_{\mathrm{eq}}^2 p_t}{\rho_c} = Q_m \quad \#(S1)$$

In this model, ∇ represents the gradient operator, ρ_c is the medium density, k_{eq}^2 is the square of the equivalent wavenumber, which includes both the wavenumber and the attenuation term, Q_m is the external energy input, and p_t is the sound pressure.

To analyze the impact of reflections at the water/PDMS interface, acoustic wave dissipation due to thermal conduction and viscosity effects is considered. The PDMS channel wall condition is set as "thermal-viscous boundary impedance." The boundary between the pressure acoustics domain and the solid mechanics domain is defined as "acoustic-solid coupling."

Next, the fluid domain in the chamber is modeled using the "Laminar Flow" module, with all walls set as no-slip boundary conditions. The "Laminar Flow" is coupled with the "Pressure Acoustics" domain through acoustic streaming. This physics is solved using a "Frequency Domain" solver at $f = 15\mathrm{MHz}$.

To effectively demonstrate the fluid reconstruction process of AVS, the "Particle Tracing for Fluid Flow" module is employed to simulate the fluid domain and solve the following motion equation:

$$\frac{\mathrm{dq}}{\mathrm{dt}} = \mathrm{u} + \frac{\tau_\mathrm{p}}{\mathrm{m_p}} F_{rad} \quad \#(S2)$$

All boundary conditions are set to "frozen," and the "acoustic streaming radiation force" is applied to the entire fluid domain. This physical field is solved using the transient solver in the particle tracing module, with output time and step sizes of 3s and 0.02s, respectively, and a relative tolerance of 1×10^{-3}.The model parameters and material parameters used in the computational study are listed in Table S1.

Note 2. Simulation of the time-averaged acoustic pressure fields

For the one-dimensional standing wave, two opposing SAWs can be generated by exciting a pair of IDTs, propagating in opposite directions on the surface of the substrate. The acoustic field in the microchannel is superposition of these waves, formulated as (*52*):

$$\mathrm{u(x,t)} = 2\sin\left[\omega \mathrm{t} + \frac{z\pi tan(\theta_R)}{\lambda_n} - \frac{2\pi z}{\lambda_f \cos(\theta_R)}\right] \cos\left(\frac{2\pi x}{\lambda_n}\right) \quad \#(S3)$$

In the equation, $u(x,t)$ represents the variation of the leaky Rayleigh wave in the fluid with respect to position x and time t, ω is the angular frequency of the wave, λ_n and λ_f are the wavelengths in the substrate and fluid, respectively. The distance between the two nodal planes is determined by the wavelength of the SAWs.

By activating two pairs of orthogonal IDTs, a grid-like 2D acoustic trap can be formed in the chamber, which is used for patterning the distribution of particles or fluid concentration. Suspended particles in the fluid under the action of acoustic waves are trapped in the Gor'kov potential well, and the acoustic radiation force F_{rad} is generated by the Gor'kov potential U, which is given by:

$$F_{rad} = -\nabla U \ \#(S4)$$

Here, U is the time-dependent Gor'kov acoustic potential, which can be calculated as follows:

$$U = \frac{4\pi r^3}{3}\left[\frac{1}{2}(\beta_f - \beta_p)\boldsymbol{p}^2 - \frac{3\rho_f(\rho_p - \rho_f)}{4\rho_p + \rho_f}\boldsymbol{v}^2\right] \ \#(S5)$$

r is the radius of a single spherical particle; p^2andv^2 are the time-averaged squares of the acoustic pressure and velocity, $\beta_f$and ρ_f are the compressibility and density of the inviscid fluid, β_p and ρ_p are the compressibility and density of the suspended particles.

The acoustic potential energy of the 2D surface standing waves in the x and y directions are respectively given by (53):

$$p_x = Asin\left(\frac{2\pi f_x}{c_n}x\right)\sin(2\pi f_x t) \ \#(S6)$$

$$p_y = Bsin\left(\frac{2\pi f_y}{c_n}y\right)\sin(2\pi f_y t) \ \#(S7)$$

In the equation, A and B are the pressure amplitudes of the standing waves in the x and y directions, respectively; f_x and f_y are the acoustic wave frequencies in the x and y directions, which are determined by the width of IDTs; c_n is the Rayleigh wave velocity in the lithium niobate substrate.

In the AVS system, since the pressure amplitudes A and acoustic wave frequencies f are the same in both the x and y directions, the time-averaged acoustic potential of the 2D acoustic field can be simplified as:

$$p_{x,y}^2 = \langle (p_x + p_y)^2 \rangle$$
$$= \frac{1}{2}[A^2 sin^2\left(\frac{2\pi f}{c_n}x\right) + A^2 sin^2\left(\frac{2\pi f}{c_n}y\right) + 2A^2 sin\left(\frac{2\pi f}{c_n}x\right) sin\left(\frac{2\pi f}{c_n}y\right)] \ \#(S8)$$

The time-averaged velocities of the 2D surface standing waves in the x and y directions are given by:

$$v_x = \frac{A}{\rho_f c_f} sin\left(\frac{2\pi f}{c_n}x\right)\sin(2\pi f t) \ \#(S9)$$

$$v_y = \frac{A}{\rho_f c_f} sin\left(\frac{2\pi f}{c_n}y\right)\sin(2\pi f t) \ \#(S10)$$

The time-averaged kinetic energy of the 2D surface standing waves is described as:

$$v_{x,y}^2 = \langle v_x^2 + v_y^2 \rangle = \frac{1}{2}(\frac{A}{\rho_f c_f})^2 \left[sin^2\left(\frac{2\pi f}{c_n}x\right) + sin^2\left(\frac{2\pi f}{c_n}y\right)\right] \#(S11)$$

By substituting the equations for the time-averaged acoustic potential and kinetic energy into Equation $(S4)$, the acoustic radiation force distribution in the 2D acoustic potential well can be derived. The corresponding equation was implemented using MATLAB software for numerical simulation, with the detailed model and material parameters provided in Table S1.(*54*)

Note 3. Calculation method of mixing index

In order to quantitatively analyze the mixing performance, images are extracted from the experimental video at the same time interval of 0.5 seconds, focusing only on the working area in the image. The gray-scale information of each pixel is analyzed, and converted into a mixing index (MI) using the following equation.

$$MI_V = \sqrt{\frac{1}{n}\sum_{i=1}^{n}\left(\frac{g_i - \bar{g}}{\bar{g}}\right)^2} \#(S12)$$

$$MI = 1 - \frac{MI_V - MI_{V,min}}{MI_{V,max} - MI_{V,min}} \#(S13)$$

Where n is the total number of pixels, g_i is the grayscale value of each pixel, and $\bar{g}$ is the average grayscale value over the n pixels. MI_V represents the variance of the grayscale values of the pixels, while $MI_{V,min}$ and $MI_{V,max}$ indicate the minimum and maximum values of MI_V, respectively. MI varies between 0 (no mixing) and 1 (complete mixing). Due to the different degrees of diffusion caused by varying flow rates, the reference standard for the maximum variance is the initial state of each time series, while the reference standard for the minimum variance is the variance value of the best mixing result.

Note 4. Evaluation of enzymatically synthesized DNA by NGS

Next-generation sequencing (NGS) was utilized to quantitatively evaluate the synthesized product. Following addition of the last cycle for each sequence, the 3' hydroxyl termini of the synthesized products were first tailed with 250 µM polyA using 1 U/µL calf thymus TdT (M0315, NEB) as per manufacturer's instructions. Subsequently, a 50 mM DTT solution containing 1% (w/v) bovine serum albumin (BSA) was injected into the chamber and incubated at 37°C for 30 minutes before retrieval. Next, the sequencing library of resulting products was prepared for dual-index paired-end sequencing analysis in two PCR steps using Phanta super-fidelity DNA polymerase (P505, Vazyme) as per manufacturer's instructions. In the first step, the polyA tailed products were amplified using universal primer with polyT (C1) and standard initiator (R1). Second, dual indices and Illumina sequencing adapters (P5 & P7) were further attached to the amplicons from the first-step PCR. After amplification, each sample was purified by VAHTS DNA clean beads (N411, Vazyme) as per manufacture's instructions and pooled in the same concentration for a shared

PE150 run (NovaSeq 6000, Illumina).

The biosynthesized DNA was evaluated primarily by error type and its rate, total yield, and average stepwise yield, which were calculated by a Perl script with the input of NGS raw data. First of all, the sequences for all synthetic oligonucleotides were extracted from the sequencing reads by a pattern matching the target region (R1-Extension Part-PolyA: /AGTGC TACTA GGACG ACTCG AATT (.*?) AAAAA AAAAA/). Subsequently, all extracted sequences were aligned to the target encoded oligonucleotide sequence, and the error types of each synthesis round were divided into three classes including deletion (DEL), insertion (INS) and substitution (SUB). After classification, the correctly synthesized bases and errors in different locations were counted. Conclusively, the error rate of each type was calculated by $N_{error}/N_{all} \times 100\%$, where N_{error} is the number of each error and N_{all} is the number of all extracted sequences multiplied by the number of synthetic rounds. By the same token, the proportion of correct bases was calculated by $N_{right}/N_{all} \times 100\%$, where N_{right} is the number of correctly synthesized bases. The full-length yield was calculated by $S_{right}/S_{all} \times 100\%$, where S_{right} is the number of correct synthetic sequences and S_{all} is the number of all extracted sequences. Average stepwise yield was calculated by $(Y_{total})^{(1/r)}$, where Y_{total} is referred to the full-length yield and r is referred to the number of synthetic rounds.

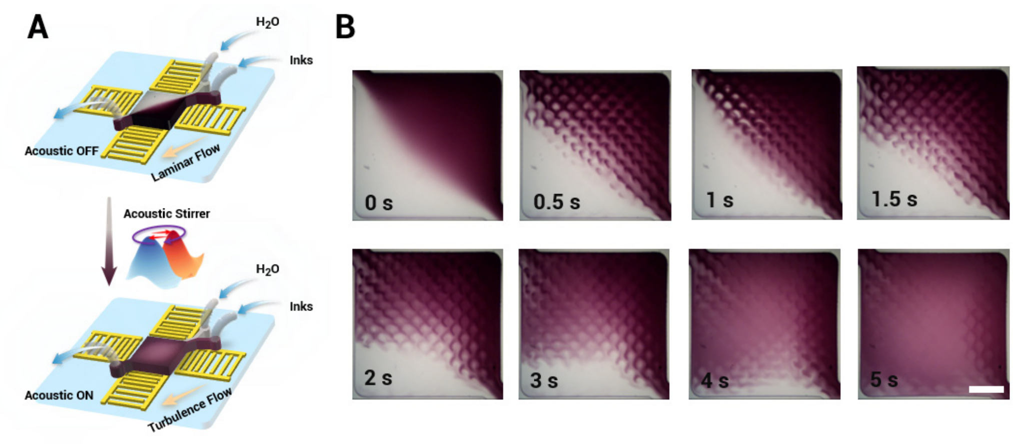

Fig. S1. Schematic diagram of AVS device.

(**A**)Schematic illustration of the experimental setup for fluid mixing using a AVS unit, which consists of two inlets and an outlet connected to a square PDMS chamber with no internal microstructure. The flow rate ratio of water to ink solution is fixed at 1:1 to ensure that the fluids have approximately the same distribution width at the cross-section. (**B**) The mixed image sequence is captured under the same observation conditions (eyepiece, exposure time, exposure gain). The fluid, which is initially in a laminar flow state, achieves good mixing and dispersion effects under the virtual stirring conditions at the center of AVS. Scale bar: 500um.

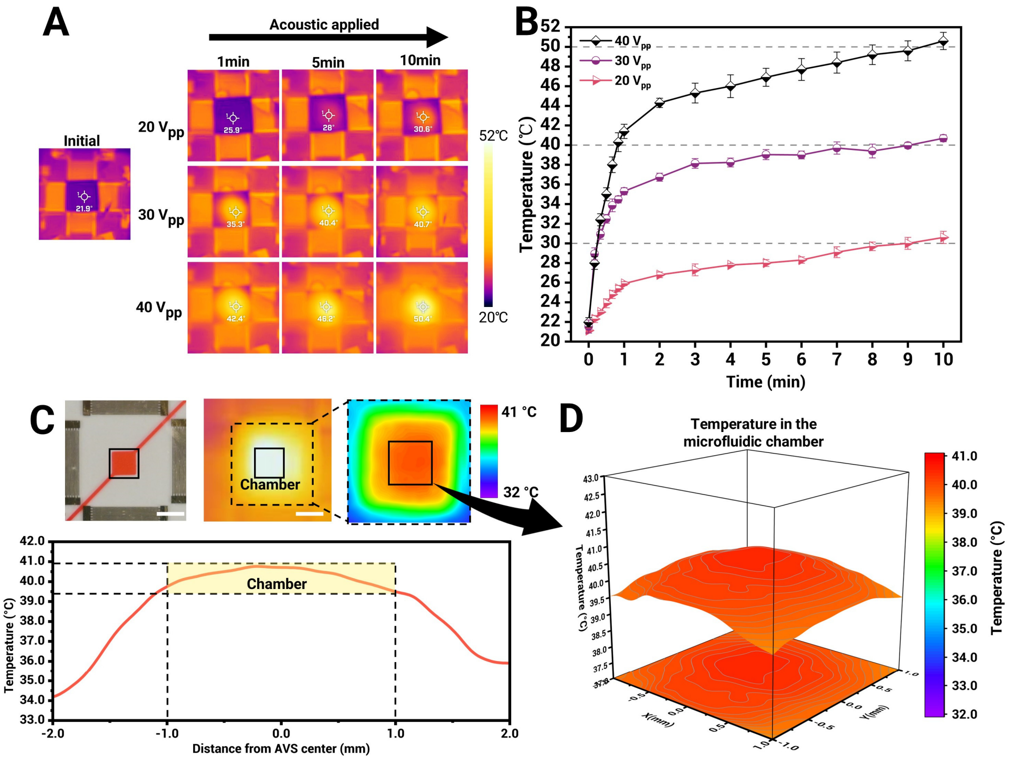

Fig. S2. Temperature distribution and uniformity of AVS.

(**A**) Photograph of infrared thermal imaging of AVS. (**B**) Thermal effect curves of AVS under input power of 20Vpp, 30Vpp, and 40Vpp. (**C**) Images of the AVS unit with PDMS microchannels filled with red ink, and the corresponding infrared thermal images. The result of device temperature distribution as a function of distance from the center of the AVS aperture is plotted below this figure. (**D**) 3D surface colormap image showing the average temperature distribution in the reaction area during 1 minute of acoustic stimulation. Scale bar: 2 mm.

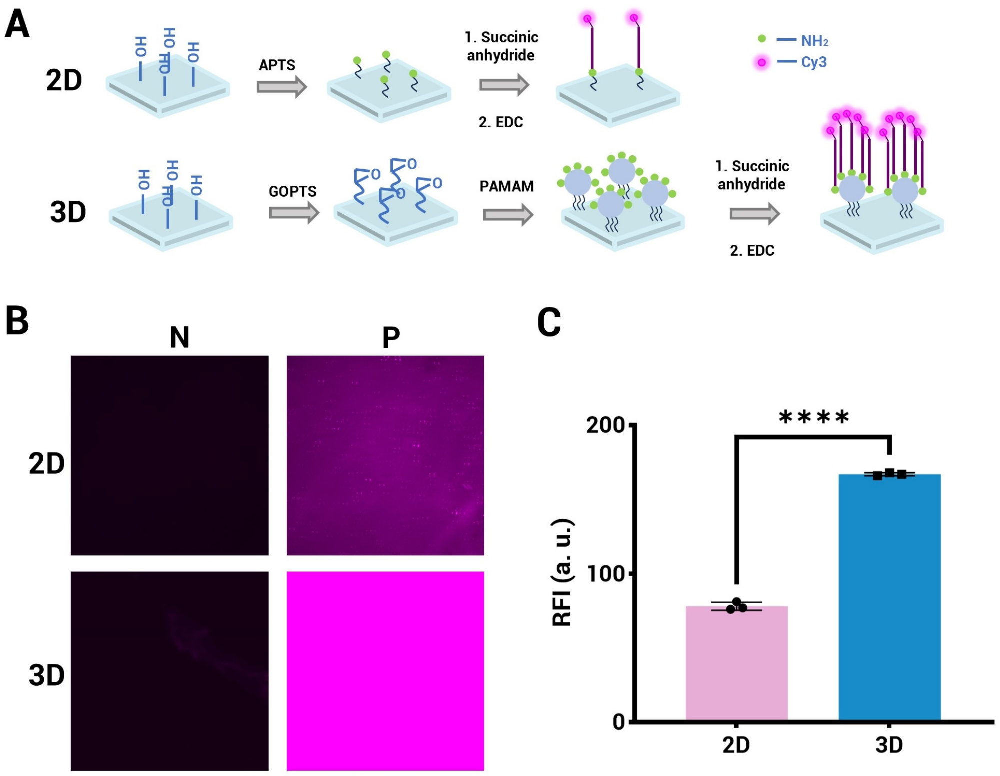

Fig. S3. Amino Modification on Lithium Niobate Substrate.

(**A**) The fabrication process for 2D amino-modified and 3D dendrimeric lithium niobate slides. (**B**) Fluorescence images of amino-modified Cy3-labeled DNA probes compared to amino-free Cy3-labeled DNA probes. (**C**) Comparison of the relative fluorescence intensity of Cy3-labeled DNA probes on 2D amino-modified and 3D dendrimeric surfaces.

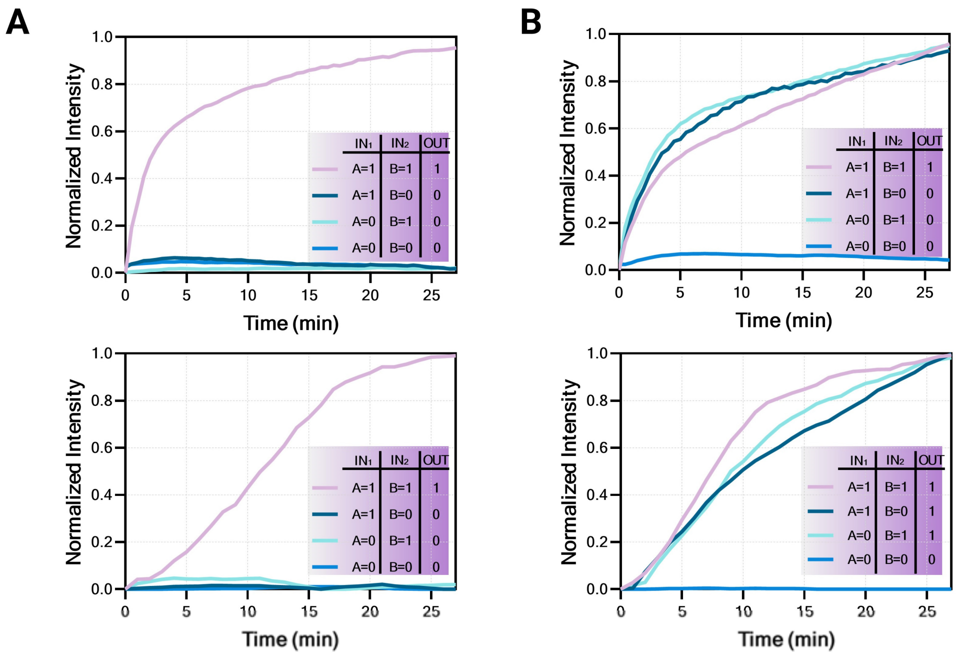

Fig. S4. Operational results of DNA logic gates on MBs, non-AVS.

(**A**) Performing DNA logic OR and AND calculations with the same system on traditional carriers involves immobilizing the gate circuit chain onto magnetic beads and introducing the reactant solution into a test tube containing these magnetic beads. The qPCR instrument is then utilized to control the reaction temperature and monitor changes in fluorescence intensity. (**B**) The results of DNA logic OR and AND calculations under non-AVS conditions.

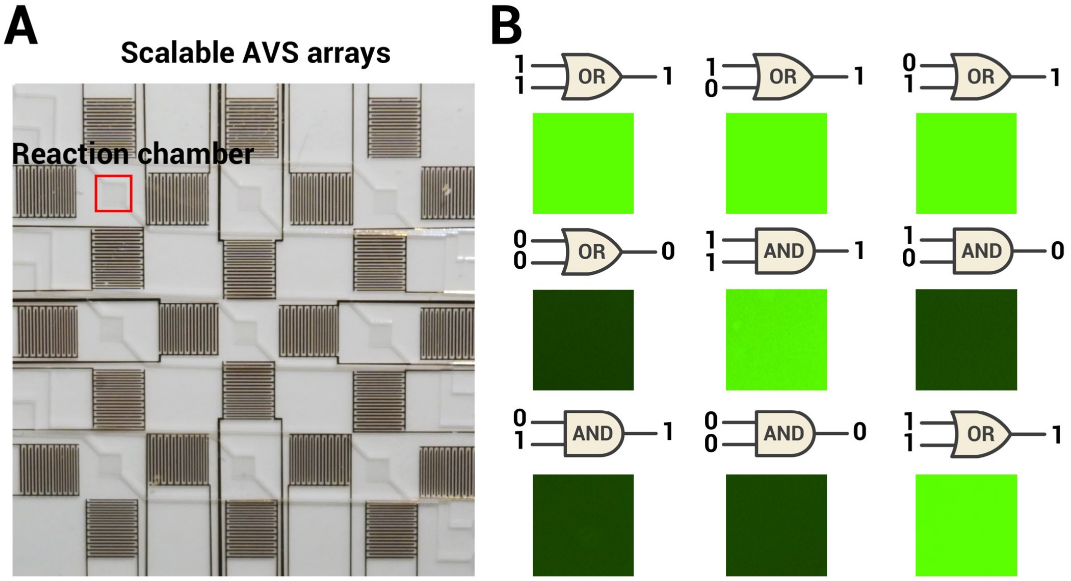

Fig. S5. Integrated parallel DNA computing based on a 3×3 AVS device.

(**A**) Photograph of scaled-up 3×3 AVS device, featuring nine parallel reaction units with independent inlets and outlets. (**B**) Fluorescence images of the nine parallel DNA Boolean logic gate computations were obtained (green: 6-FAM). It's worth noting that there are a total of 8 different calculations, with one identical input condition repeated twice."

Table S1. Parameters used in simulation model. For the numerical simulation and finite element method based simulation, the parameters of materials are provided.

Symbol	parameters	Value
β_f	Compressibility of water	$4.6 \times 10^{-10}\ Pa^{-1}$
ρ_f	Density of water	$1{,}000\ kg \cdot m^{-3}$
β_p	Compressibility of the suspended particle	$2.2 \times 10^{-10}\ Pa^{-1}$
ρ_p	Density of the suspended particle	$1{,}065\ kg \cdot m^{-3}$
r	Radius	$1\ \mu m$
c_n	Sound speed in the lithium niobate substrate	$3{,}960\ m \cdot s^{-1}$
c_f	Sound speed in water	$1{,}483\ m \cdot s^{-1}$
f	Frequency of SAWs	15 Mhz
μ	Viscosity of water	$1 \times 10^{-3}\ Pa \cdot s$
d	Width of Interdigitated Transducer (IDT)	$65\ \mu m$
w	Width of PDMS channel	$900\ \mu m$
h	Height of PDMS channel	$100\ \mu m$

Table S2. DNA sequences used for experiments in this work. The DNA sequences used in different experiments are provided.

Name	Length (nt)	5’	3’	Sequence (5’ to 3’)
R1	18		NH2 C6	ACTAGGACGACTCGAATT
R1’	18		Cy3	AATTCGAGTCGTCCTAGT
Tail Adapter	20	P	6-FAM	CGACT GAACC CAAGC AACTG
Splint	38			CAG TTG CTT GGG TTC AGT CGA ATT CGA GTC GTC CTA GT
Input_A	30			CACCATTACAATCCACAATCCACACTT CCA
Input_B	30			CATCTACAATTCACACACCAATACTCC TCA
Input_C/Fuel	30			CACTATACACACCCACAACCCTAACC ATCA
Gate_1	90	NH2 C6		TGAAATGTAGGAATGTGGAGGTAATG AATG TGATGGTTAGGGTTGTGGGTGTGTAT AGTG TGGAAGTGTGGATTGTGGATTGTAAT GGTG
Gate_2	90	NH2 C6		TGAAATGTAGGAATGTGGAGGTAATG AATG TGATGGTTAGGGTTGTGGGTGTGTAT AGTG TGAGGAGTATTGGTGTGTGAATTGTA GATG
Gate_3	90	NH2 C6		TGAAATGTAGGAATGTGGAGGTAATG AATG TGGAAGTGTGGATTGTGGATTGTAAT GGTG TGATGGTTAGGGTTGTGGGTGTGTAT AGTG
Reporter_F	62	6-FAM		CGTGTGATTGTGTTATGTGGGATGAA GATTTG TGAAATGTAGGAATGTGGAGGTAATG AATG
Reporter_Q	32		BHQ1	CAAATCTTCATCCCACATAACACAAT CACACG
Output	30			CATTCATTACCTCCACATTCCTACATT TCA

Movie S1. The dynamic oscillating of particles by AVS.
Fluorescent microspheres were used to demonstrate the oscillation process of AVS. As the frequency of AVS increased, the microsphere array also oscillated at a faster speed.

Movie S2. The dynamic fluid mixing process by AVS.
Two-phase continuous flow is mixed rapidly when AVS arrays are applied.

REFERENCES AND NOTES

1. P.-S. Huang, S. E. Boyken, D. Baker, The coming of age of de novo protein design. *Nature* **537**, 320–327 (2016).

2. M. Hönig, P. Sondermann, N. J. Turner, E. M. Carreira, Enantioselective chemo- and biocatalysis: Partners in retrosynthesis. *Angew. Chem. Int. Ed. Engl.* **56**, 8942–8973 (2017).

3. S. Palluk, D. H. Arlow, T. de Rond, S. Barthel, J. S. Kang, R. Bector, H. M. Baghdassarian, A. N. Truong, P. W. Kim, A. K. Singh, N. J. Hillson, J. D. Keasling, De novo DNA synthesis using polymerase-nucleotide conjugates. *Nat. Biotechnol.* **36**, 645–650 (2018).

4. Y. Dong, F. Sun, Z. Ping, Q. Ouyang, L. Qian, DNA storage: Research landscape and future prospects. *Natl. Sci. Rev.* **7**, 1092–1107 (2020).

5. H. Su, J. Xu, Q. Wang, F. Wang, X. Zhou, High-efficiency and integrable DNA arithmetic and logic system based on strand displacement synthesis. *Nat. Commun.* **10**, 5390 (2019).

6. H. Lv, N. Xie, M. Li, M. Dong, C. Sun, Q. Zhang, L. Zhao, J. Li, X. Zuo, H. Chen, F. Wang, C. Fan, DNA-based programmable gate arrays for general-purpose DNA computing. *Nature* **622**, 292–300 (2023).

7. D. Verardo, B. Adelizzi, D. A. Rodriguez-Pinzon, N. Moghaddam, E. Thomee, T. Loman, X. Godron, A. Horgan, Multiplex enzymatic synthesis of DNA with single-base resolution. *Sci. Adv.* **9**, eadi0263 (2023).

8. L. Yang, Q. Tang, M. Zhang, Y. Tian, X. Chen, R. Xu, Q. Ma, P. Guo, C. Zhang, D. Han, A spatially localized DNA linear classifier for cancer diagnosis. *Nat. Commun.* **15**, 4583 (2024).

9. C. N. Takahashi, B. H. Nguyen, K. Strauss, L. Ceze, Demonstration of end-to-end automation of DNA data storage. *Sci. Rep.* **9**, 4998 (2019).

10. A. J. deMello, Control and detection of chemical reactions in microfluidic systems. *Nature* **442**, 394–402 (2006).

11. H. Pei, X. Zuo, D. Zhu, Q. Huang, C. Fan, Functional DNA nanostructures for theranostic applications. *Acc. Chem. Res.* **47**, 550–559 (2014).

12. E. A. Josephs, T. Ye, A single-molecule view of conformational switching of DNA tethered to a gold electrode. *J. Am. Chem. Soc.* **134**, 10021–10030 (2012).

13. M. Bayareh, M. N. Ashani, A. Usefian, Active and passive micromixers: A comprehensive review. *Chem. Eng. Process. Process Intensif.* **147**, 107771 (2020).

14. M. Rafeie, M. Welleweerd, A. Hassanzadeh-Barforoushi, M. Asadnia, W. Olthuis, M. Ebrahimi Warkiani, An easily fabricated three-dimensional threaded lemniscate-shaped micromixer for a wide range of flow rates. *Biomicrofluidics* **11**, 014108 (2017).

15. L. Yang, F. Xu, G. Chen, Effective mixing in a passive oscillating micromixer with impinging jets. *Chem. Eng. J.* **489**, 151329 (2024).

16. D. Lee, P.-H. Lo, On the enhancement of mixing in tangentially crossing micro-channels. *Chem. Eng. J.* **181-182**, 524–529 (2012).

17. S. Wang, K. Liu, J. Liu, Z. T.-F. Yu, X. Xu, L. Zhao, T. Lee, E. K. Lee, J. Reiss, Y.-K. Lee, L. W. K. Chung, J. Huang, M. Rettig, D. Seligson, K. N. Duraiswamy, C. K.-F. Shen, H.-R. Tseng, Highly efficient capture of circulating tumor cells by using nanostructured silicon substrates with integrated chaotic micromixers. *Angew. Chem. Int. Ed. Engl.* **123**, 3140–3144 (2011).

18. S. Hossain, A. Fuwad, K.-Y. Kim, T.-J. Jeon, S. M. Kim, Investigation of mixing performance of two-dimensional micromixer using tesla structures with different shapes of obstacles. *Ind. Eng. Chem. Res.* **59**, 3636–3643 (2020).

19. H. Song, Z. Cai, H. Noh, D. J. Bennett, Chaotic mixing in microchannels via low frequency switching transverse electroosmotic flow generated on integrated microelectrodes. *Lab Chip* **10**, 734–740 (2010).

20. K.-R. Huang, J.-S. Chang, S. D. Chao, T.-S. Wung, K.-C. Wu, Study of active micromixer driven by electrothermal force. *Jpn. J. Appl. Phys.* **51**, 047002 (2012).

21. D. Owen, M. Ballard, A. Alexeev, P. J. Hesketh, Rapid microfluidic mixing via rotating magnetic microbeads. *Sens. Actuators A Phys.* **251**, 84–91 (2016).

22. S. Feng, C. Pan, H. Ye, W. Liu, W. Yang, Y. Lv, S. Tao, Magnetic non-spherical particles inducing vortices in microchannel for effective mixing. *Small* **19**, 2207383 (2023).

23. J. Rufo, F. Cai, J. Friend, M. Wiklund, T. J. Huang, Acoustofluidics for biomedical applications. *Nat. Rev. Methods Primers* **2**, 30 (2022).

24. R. Zhong, M. Sullivan, N. Upreti, R. Chen, A. De Ganzó, K. Yang, S. Yang, K. Jin, Y. He, K. Li, J. Xia, Z. Ma, L. P. Lee, T. Konry, T. J. Huang, Cellular immunity analysis by a modular acoustofluidic platform: CIAMAP. *Sci. Adv.* **9**, eadj9964 (2023).

25. Y. Yang, L. Zhang, K. Jin, M. He, W. Wei, X. Chen, Q. Yang, Y. Wang, W. Pang, X. Ren, X. Duan, Self-adaptive virtual microchannel for continuous enrichment and separation of nanoparticles. *Sci. Adv.* **8**, eabn8440 (2022).

26. J. Xia, Z. Wang, R. Becker, F. Li, F. Wei, S. Yang, J. Rich, K. Li, J. Rufo, J. Qian, K. Yang, C. Chen, Y. Gu, R. Zhong, P. J. Lee, D. T. W. Wong, L. P. Lee, T. J. Huang, Acoustofluidic virus isolation via bessel beam excitation separation technology. *ACS Nano* **18**, 22596–22607 (2024).

27. T. Li, J. Li, L. Bo, H. Bachman, B. Fan, J. Cheng, Z. Tian, Robot-assisted chirality-tunable acoustic vortex tweezers for contactless, multifunctional, 4-DOF object manipulation. *Sci. Adv.* **10**, eadm7698 (2024).

28. X. Xu, K. Jin, K. Yang, R. Zhong, M. Liu, W. Collyer, S. Jain, Y. Chen, J. Xia, J. Li, S. Yang, E. H. Dowell, T. J. Huang, Acoustofluidic tweezers via ring resonance. *Sci. Adv.* **10**, eads2654 (2024).

29. S. Pan, T. Jeon, D. C. Luther, X. Duan, V. M. Rotello, Cytosolic delivery of functional proteins *in vitro* through tunable gigahertz acoustics. *ACS Appl. Mater. Interfaces* **12**, 15823–15829 (2020).

30. J. Xu, H. Cai, Z. Wu, X. Li, C. Tian, Z. Ao, V. C. Niu, X. Xiao, L. Jiang, M. Khodoun, M. Rothenberg, K. Mackie, J. Chen, L. P. Lee, F. Guo, Acoustic metamaterials-driven transdermal drug delivery for rapid and on-demand management of acute disease. *Nat. Commun.* **14**, 869 (2023).

31. X. Gao, X. Hu, J. Zheng, Q. Hu, S. Zhao, L. Chen, Y. Yang, On-demand liquid microlens arrays by non-contact relocation of inhomogeneous fluids in acoustic fields. *Lab Chip* **22**, 3942–3951 (2022).

32. Q. Yin, Y. Luo, X. Yu, K. Chen, W. Li, H. Huang, L. Zhang, Y. Zhou, B. Zhu, Z. Ma, W. Zhang, Acoustic cell patterning for structured cell-laden hydrogel fibers/tubules. *Adv. Sci.* **11**, 2308396 (2024).

33. Z. Wang, J. Rich, N. Hao, Y. Gu, C. Chen, S. Yang, P. Zhang, T. J. Huang, Acoustofluidics for simultaneous nanoparticle-based drug loading and exosome encapsulation. *Microsyst. Nanoeng.* **8**, 45 (2022).

34. Y. Lu, W. Tan, S. Mu, G. Zhu, Vortex-enhanced microfluidic chip for efficient mixing and particle capturing combining acoustics with inertia. *Anal. Chem.* **96**, 3859–3869 (2024).

35. X. Hu, J. Zheng, Q. Hu, L. Liang, D. Yang, Y. Cheng, S.-S. Li, L.-J. Chen, Y. Yang, Smart acoustic 3D cell construct assembly with high-resolution. *Biofabrication* **14**, 045003 (2022).

36. X. Hu, J. Zheng, Q. Zhu, Q. Wu, S.-S. Li, Y. Yang, L.-J. Chen, Acoustic assembly and scanning of superlens arrays for high-resolution and large field-of-view bioimaging. *ACS Nano* **18**, 15218–15228 (2024).

37. F. Guo, Z. Mao, Y. Chen, Z. Xie, J. P. Lata, P. Li, L. Ren, J. Liu, J. Yang, M. Dao, S. Suresh, T. J. Huang, Three-dimensional manipulation of single cells using surface acoustic waves. *Proc. Natl. Acad. Sci. U.S.A.* **113**, 1522–1527 (2016).

38. J. T. Karlsen, H. Bruus, Acoustic tweezing and patterning of concentration fields in microfluidics. *Phys. Rev. Applied* **7**, 034017 (2017).

39. J. T. Karlsen, P. Augustsson, H. Bruus, Acoustic force density acting on inhomogeneous fluids in acoustic fields. *Phys. Rev. Lett.* **117**, 114504 (2016).

40. X. Gao, D. Li, S. Zhao, D. Yang, Q. Wu, S.-S. Li, L. Zhang, L.-J. Chen, Y. Yang, X. Hu, Acoustic controllable spatiotemporal cell micro-oscillation for noninvasive intracellular drug delivery. *Anal. Chem.* **96** 14998–15007 (2024).

41. X. Liu, N. Rong, Z. Tian, J. Rich, L. Niu, P. Li, L. Huang, Y. Dong, W. Zhou, P. Zhang, Y. Chen, C. Wang, L. Meng, T. J. Huang, H. Zheng, Acoustothermal transfection for cell therapy. *Sci. Adv.* **10**, eadk1855 (2024).

42. I. W. Sizer, "Effects of temperature on enzyme kinetics" in *Advances in Enzymology and Related Areas of Molecular Biology*, F. F. Nord, C. H. Werkman, Eds. (Wiley, ed. 1, 1943) vol. **3**, pp. 35–62.

43. J. C. Aledo, S. Jiménez-Riveres, M. Tena, The effect of temperature on the enzyme-catalyzed reaction: Insights from thermodynamics. *J. Chem. Educ.* **87**, 296–298 (2010).

44. A. G. Marangoni, "Characterization of enzyme stability" in *Enzyme Kinetics*, A. G. Marangoni, Ed. (Wiley, ed. 1, 2002), pp. 140–157.

45. C. He, J. Yao, C. Yang, J. Wang, B. Sun, G. Liao, T. Shi, Z. Liu, Irreversible bonding of polydimethylsiloxane-lithium niobate using oxygen plasma modification for surface acoustic wave based microfluidic application: Theory and experiment. *Small Methods* **8**, e2301321 (2024).

46. J. Cao, Z. Zheng, D. Sun, X. Chen, R. Cheng, T. Lv, Y. An, J. Zheng, J. Song, L. Wu, C. Yang, Decoder-seq enhances mRNA capture efficiency in spatial RNA sequencing. *Nat. Biotechnol.* **42**, 1735–1746 (2024).

47. X. Lu, J. Li, C. Li, Q. Lou, K. Peng, B. Cai, Y. Liu, Y. Yao, L. Lu, Z. Tian, H. Ma, W. Wang, J. Cheng, X. Guo, H. Jiang, Y. Ma, Enzymatic DNA synthesis by engineering terminal deoxynucleotidyl transferase. *ACS Catal.* **12**, 2988–2997 (2022).

48. K. Li, X. Lu, J. Liao, H. Chen, W. Lin, Y. Zhao, D. Tang, C. Li, Z. Tian, Z. Zhu, H. Jiang, J. Sun, H. Zhang, C. Yang, DNA-DISK: Automated end-to-end data storage via enzymatic single-nucleotide DNA synthesis and sequencing on digital microfluidics. *Proc. Natl. Acad. Sci. U.S.A.* **121**, e2410164121 (2024).

49. T. Song, A. Eshra, S. Shah, H. Bui, D. Fu, M. Yang, R. Mokhtar, J. Reif, Fast and compact DNA logic circuits based on single-stranded gates using strand-displacing polymerase. *Nat. Nanotechnol.* **14**, 1075–1081 (2019).

50. N. R. Y. Ho, G. S. Lim, N. R. Sundah, D. Lim, T. P. Loh, H. Shao, Visual and modular detection of pathogen nucleic acids with enzyme–DNA molecular complexes. *Nat. Commun.* **9**, 3238 (2018).

51. N. R. Sundah, A. Natalia, Y. Liu, N. R. Y. Ho, H. Zhao, Y. Chen, Q. H. Miow, Y. Wang, D. L. L. Beh, K. L. Chew, D. Chan, P. A. Tambyah, C. W. M. Ong, H. Shao, Catalytic amplification by transition-state molecular switches for direct and sensitive detection of SARS-CoV-2. *Sci. Adv.* **7**, eabe5940 (2021).

52. F. Guo, P. Li, J. B. French, Z. Mao, H. Zhao, S. Li, N. Nama, J. R. Fick, S. J. Benkovic, T. J. Huang, Controlling cell–cell interactions using surface acoustic waves. *Proc. Natl. Acad. Sci. U.S.A.* **112**, 43–48 (2015).

53. Z. Hou, Z. Zhou, P. Liu, Y. Pei, Deformable oscillation of particles patterning by parametric bulk acoustic waves. *Extreme Mech. Lett.* **37**, 100716 (2020).

54. S. Yang, Z. Tian, Z. Wang, J. Rufo, P. Li, J. Mai, J. Xia, H. Bachman, P.-H. Huang, M. Wu, C. Chen, L. P. Lee, T. J. Huang, Harmonic acoustics for dynamic and selective particle manipulation. *Nat. Mater.* **21**, 540–546 (2022).
